# Oregano extract induces apoptosis and inhibits autophagy in HepG2 cells via the PI3K/AKT pathway

**DOI:** 10.3389/fphar.2026.1884083

**Published:** 2026-07-16

**Authors:** Minglu Xu, Miaomiao Zhu, Wen-yu Zhao, Zhenwei Li, Kai Xu, Kun Yang, Zhixia Wang, Siqi Long, Yingying Zhang, Ying Cao, Shuang Huang, Zhongzheng Li, Guoying Yu, Lan Wang

**Affiliations:** 1 School of Chemistry and Chemical Engineering, Henan Institute of Science and Technology, Xinxiang, Henan, China; 2 State Key Laboratory of Cell Differentiation and Regulation, Henan International Joint Laboratory of Pulmonary Fibrosis, Henan Center for Outstanding Overseas Scientists of Organ Fibrosis, Institute of Biomedical Science, College of Life Science, Henan Normal University, Xinxiang, China; 3 Department of Pulmonary and Critical Care Medicine, The First Affiliated Hospital of Xinxiang Medical University, Weihui, Henan, China

**Keywords:** apoptosis, autophagy, ethyl acetate layer extract of oregano, liver cancer, PI3K/AKT pathway

## Abstract

Hepatocellular carcinoma (HCC) remains one of the most common and highly lethal malignancies globally. Conventional therapeutic approaches are often limited by suboptimal efficacy and significant toxicity, driving the search for alternative strategies, particularly those derived from natural products. This study aimed to evaluate the antitumor activity and underlying molecular mechanisms of the ethyl acetate extract of “Origanum vulgare” L (EAO) in human hepatocellular carcinoma cells. Our findings demonstrate that EAO markedly suppressed HepG2 and Huh7 cell proliferation and promoted apoptosis, accompanied by increased reactive oxygen species (ROS) generation, loss of mitochondrial membrane potential, and ATP depletion. Additionally, EAO inhibited autophagy, as indicated by decreased LC3-II expression and increased p62 accumulation. Mechanistic investigations revealed that EAO inactivated the PI3K/AKT signaling pathway, and these effects were reversed by the ROS scavenger N-acetylcysteine (NAC), confirming ROS-dependent suppression of this pathway. Both network pharmacology and RNA-sequencing analyses further supported PI3K/AKT as a critical regulatory node. In summary, EAO exerts potent antitumor effects against hepatocellular carcinoma by inducing mitochondrial dysfunction and apoptosis, while concurrently suppressing autophagy through ROS-mediated inhibition of the PI3K/AKT pathway. These results highlight EAO as a promising low-toxicity candidate for the treatment of liver cancer.

## Introduction

Hepatocellular carcinoma (HCC) is the sixth most common cancer worldwide and the third leading cause of cancer-related mortality, with over 850,000 new cases diagnosed annually (Xing et al., 2023; Torre et al., 2015; Zhou et al., 2025). Despite advances in early detection and targeted therapies, the prognosis for HCC remains poor due to high recurrence and limited treatment options for advanced-stage patients (Chen et al., 2019; Liu et al., 2019; Wu et al., 2023). Conventional treatments such as surgical resection, radiofrequency ablation, chemotherapy, and molecular-targeted therapies have only modestly improved outcomes, partly because of high recurrence rates, emerging drug resistance, and significant side effects (Yang et al., 2022; Anderson et al., 2019). This situation underscores the urgent need for novel therapeutic strategies and adjuvant treatments.

In recent years, natural products have attracted considerable attention in cancer therapy due to their multi-targeted actions and relatively low toxicity (Xie et al., 2024). Traditional herbal medicines often contain a complex mixture of bioactive compounds capable of modulating key cellular processes such as proliferation, apoptosis, cell cycle progression, and autophagy (Fikry et al., 2019). For example, compounds like matrine and apigenin have demonstrated the ability to inhibit cancer cell invasion and promote apoptosis by modulating signaling pathways, including NF-κB and PI3K/AKT/mTOR (Suntres et al., 2015). The structural diversity and multifaceted pharmacological activities of natural products make them promising candidates for multi-targeted therapy, potentially reducing the risk of drug resistance (Abusaliya et al., 2023; Chen et al., 2023).

Origanum vulgare (oregano) is a traditional medicinal herb that has long been used in traditional medicine, where the whole plant is employed for therapeutic purposes. Recent studies have revealed that oregano extract (EAO) is rich in bioactive constituents—primarily carvacrol and various flavonoids (Sharifi-Rad et al., 2018; Sarac and Ugur, 2008). Carvacrol has been shown to induce apoptosis, regulate the cell cycle, and interfere with cancer cell metabolism in several tumor models. Meanwhile, flavonoids often exert anticancer effects by generating reactive oxygen species (ROS), which activate intrinsic apoptotic and autophagic pathways (Zhou et al., 2016; Aune et al., 2017; Kayagaki et al., 2024). The synergistic interaction of these compounds in oregano extract provides a solid theoretical basis for its potential application in anticancer therapy (Sarac and Ugur, 2008).

Despite these promising findings, systematic research on the anticancer effects of oregano extract in HCC and its underlying molecular mechanisms remains limited (Torre et al., 2015). Although some *in vitro* studies have demonstrated significant anti-proliferative and pro-apoptotic effects of carvacrol and flavonoids in various cancer models, investigations specifically addressing their role in HCC—particularly in human liver cancer cell lines such as HepG2—are still insufficient (Hu et al., 2015; Yang et al., 2018; Tian et al., 2019). The specific molecular pathways through which oregano extract modulates cell proliferation, induces apoptosis, and activates autophagy in HCC cells have not been fully elucidated (Ma et al., 2016).

Given this background, the present study aims to investigate the inhibitory effects of oregano extract on the proliferation of HepG2 and Huh7 cells. The potential molecular mechanisms underlying its ability to induce apoptosis and autophagy will be further explored. Clarifying the synergistic anticancer mechanisms of the bioactive compounds in oregano extract is expected to provide novel theoretical support and experimental evidence for the application of natural products in HCC therapy and the development of new, low-toxicity anticancer agents.

## Materials and methods

### Chemicals and reagents

Dulbecco’s Modified Eagle Medium (DMEM), RPMI-1640, DMEM/F-12, 100 IU/ml streptomycin, 100 IU/ml penicillin, and fetal bovine serum (FBS) were purchased from Thermo Fisher Scientific and Gibco. The EdU cell proliferation assay kit was obtained from RiboBio (Guangzhou, China). Hoechst 33258, Annexin V-FITC/PI apoptosis detection kit, and propidium iodide (PI) were procured from Solarbio (Beijing, China). Cell Counting Kit-8 (CCK-8), Reactive Oxygen Species (ROS) Assay Kit, mitochondrial membrane potential assay kit with JC-1, and caspase-3 activity detection kit were purchased from Beyotime (Guangzhou, China). Primary antibodies, including Cleaved Caspase-3, Caspase-3, AKT, p-AKT, MFN1, MFN2, DRP1, and p-DRP1, were obtained from Cell Signaling Technology (Shanghai, China). Antibodies for Bax, Bcl-2, Beclin-1, OPA1, FIS1, and GAPDH were purchased from ProteinTech (Wuhan, China). Antibodies for PI3K, p-PI3K, and LC3B were obtained from Abcam (Shanghai, China).

### Cell culture

The human hepatocyte cell line HL-7702 and the liver cancer cell line HepG2 and Huh7 were purchased from the Shanghai Cell Resource Center (Shanghai, China). The A549 lung carcinoma cell line, RD rhabdomyosarcoma cell line, and HuT78 cutaneous T-cell lymphoma cell line were obtained from the American Type Culture Collection (ATCC, Shanghai, China). HepG2 and Huh7 cells were cultured in Dulbecco’s Modified Eagle Medium (DMEM) supplemented with 10% (v/v) fetal bovine serum (FBS), 100 IU/ml penicillin, and 100 μg/ml streptomycin at 37 °C in a humidified incubator with 5% CO_2_. HL-7702, RD, and HuT78 cells were maintained in Roswell Park Memorial Institute (RPMI)-1640 medium supplemented with 10% FBS, 100 IU/ml penicillin, and 100 μg/ml streptomycin under the same culture conditions. A549 cells were cultured in DMEM/F-12 medium supplemented with 10% FBS, 100 IU/ml penicillin, and 100 μg/ml streptomycin at 37 °C with 5% CO_2_.

### Cell viability assay

HepG2 cells were seeded at a density of approximately 3 × 10^3^ cells per well in a 96-well plate and allowed to adhere overnight. Cells were treated with different concentrations of oregano extract (EAO) for the indicated time periods. After treatment, the culture medium was discarded, and 90 μL of fresh complete medium, along with 10 μL of Cell Counting Kit-8 (CCK-8) reagent, was added to each well. The plate was gently mixed and incubated at 37 °C in the dark for 1 h. Following incubation, the absorbance at 450 nm was measured using a microplate reader.

### Colony formation assay

HepG2 and Huh7 cells were counted and seeded in a 6-well culture plate at a density of 1 × 10^3^ cells per well. After adherence, cells were treated with different concentrations of EAO (0, 50, and 100 μg/mL) for 48 h. The culture medium was replaced every 7 days, and the cells were allowed to grow for 14 days. After the incubation period, cells were fixed with 4% paraformaldehyde (PFA) for 20 min at room temperature and washed twice with PBS. The fixed cells were stained with 0.5% crystal violet for 30 min at room temperature, washed with PBS, and air-dried. Colonies were photographed and counted under a microscope.

### EdU detection

After treatment with EAO, cells were incubated with 50 μM EdU medium for 2 h at 37 °C. After incubation, cells were fixed with 4% PFA for 20 min at room temperature. The cells were then treated with 1×Apollo staining reaction solution according to the manufacturer’s instructions, followed by staining with 1×Hoechst 33342 reaction solution for nuclear labeling. Finally, the cells were washed three times with PBS, and images were acquired using a fluorescence microscope.

### Cell cycle detection

After treatment with the indicated EAO, cells were harvested and washed twice with PBS. The cells were then fixed with cold 70% (v/v) ethanol at 4 °C for 24 h. Following fixation, the cells were washed with cold PBS and resuspended in PBS containing 0.05 mg/mL RNase A and 25 μg/mL propidium iodide (PI). The mixture was incubated at 37 °C for 15 min in the dark. After incubation, the cells were filtered through a mesh sieve to remove aggregates. The cell cycle distribution was analyzed using flow cytometry.

### Cell morphology and nuclear observation

HepG2 cells were seeded into 24-well plates at a density of 1 × 10^4^ cells per well and incubated overnight for attachment. The cells were then treated with different concentrations of the extract for 48 h. After treatment, the cells were stained with 1×Hoechst 33342 solution at room temperature for 15 min in the dark. The cells were washed three times with PBS, and the nuclear morphology was observed under a fluorescence microscope.

### Annexin V and PI staining

Cell apoptosis was assessed using the Annexin V-FITC/PI apoptosis kit. Briefly, approximately 1 × 10^5^ cells per sample were harvested after the indicated treatment and washed twice with PBS. Each sample was then resuspended in 500 μL of 1× binding buffer, followed by incubation with 5 μL Annexin V and 10 μL PI for 15 min at room temperature in the dark. After staining, the samples were analyzed by flow cytometry using a FACS Calibur flow cytometer (BD Immunocytometry Systems, USA).

### Analysis of Caspase-3 activity

HepG2 and Huh7 cells were seeded in 96-well plates and treated with EAO for 48 h. An untreated group was used as the negative control. After treatment, the cells were incubated with a medium containing 5 μM GreenNuc™ Caspase-3 Substrate for 30 min in an incubator. Caspase-3 activity was measured using a fluorescent plate reader, detecting the signal in the green fluorescence channel.

### Autophagy detection

HepG2 cells (3 × 10^4^cells per well) were seeded in 24-well plates. Once the cells reached approximately 80% confluence, they were transfected with the GFP-RFP-LC3 plasmid using Lipofectamine 3000 according to the manufacturer’s protocol. After 24 h, the treatment group was exposed to 84 μg/mL of EAO. Following another 24 h of EAO treatment, chloroquine (50 μM) was added, and cells were incubated for 4 h. The cells were then fixed with 4% paraformaldehyde and stained with DAPI. Autophagy was assessed by fluorescence microscopy (Zeiss, Germany), and the images were analyzed.

### Network pharmacological analysis of liver cancer by EAO

The network pharmacological analysis of EAO in liver cancer was conducted using a systematic approach. First, potential targets were predicted by searching the keywords “liver cancer” and “liver carcinoma” in GeneCards, UALCAN, and DisGeNET databases. The collected liver cancer-related genes were then intersected with the targets of active ingredients in EAO using an online tool (available at: http://www.bioinformatics.com.cn/static/others/jvenn/index.html). Gene Ontology (GO) functional enrichment analysis and Kyoto Encyclopedia of Genes and Genomes (KEGG) pathway enrichment analysis were performed using the ClusterProfiler package in R, with *Homo sapiens* set as the species and a significance threshold of *p* < 0.05. The enriched functions and pathways were then visualized. To explore protein interactions, the potential targets of EAO were imported into the STRING database with a high-confidence threshold of 0.700, followed by PPI network construction in Cytoscape. Finally, a “Component-Target-Pathway” network was established in Cytoscape by integrating data from enrichment analyses, potential target genes, and major related pathways, providing a comprehensive visualization of EAO’s molecular mechanisms in liver cancer.

### RNA-seq analysis of HepG2 cells

For RNA-Seq analysis, HepG2 cells were seeded in a dish and treated with EAO at its IC_50_ concentration (84.42 μg/mL) for 24 h. After treatment, approximately 2 million cells were collected, and total RNA was extracted using Trizol reagent before being sent for RNA sequencing. Differential gene expression analysis was conducted using the limma package in R, with significance thresholds set at *p* < 0.05 and |log2Fold Change| > 0.584 to identify up- and downregulated genes for further analysis. Additionally, Gene Set Enrichment Analysis (GSEA) was performed using the ClusterProfiler package and the molecular signature database V7.4, which includes the hallmark (H) and ontology (C5) gene sets. The KEGG dataset was selected for human species, and pathways with *p* < 0.05 were considered significantly enriched.

### RNA-seq library construction, sequencing and bioinformatic analysis

Total RNA isolated from all samples was used to construct strand-specific RNA-seq libraries, which were sequenced on an Illumina platform to generate 15–20 million paired-end raw reads per sample. Raw FASTQ reads were subjected to quality assessment using FastQC and trimmed with Trimmomatic, and only clean reads with Q20 > 97% and Q30 > 92% were retained. Qualified reads were aligned to the reference genome via STAR, with overall mapping rates ranging from 86% to 93% and uniquely mapped reads accounting for more than 80% of total clean reads. The gene raw count matrix was imported into DESeq2 for differential expression analysis. Raw P values derived from Wald tests were adjusted by the Benjamini–Hochberg procedure for false discovery rate (FDR) control, and genes with FDR <0.05 and |log_2_ (fold change)| ≥ 1 were regarded as differentially expressed genes. All raw sequencing reads, alignment files and processed expression matrices have been deposited in the Genome Sequence Archive (GSA) of the China National Center for Bioinformation (CNCB) under the accession PRJCA067345.

### ROS level measurement

DCFH-D reagent was diluted with serum-free medium and added to the treated cell wells. The cells were incubated with the reagent for 20 min. After incubation, the fluorescence intensity was measured using a green light filter on a fluorescent enzyme labeling reader.

### ATP level determination

Following treatment, 100 μL of cell culture supernatant and 100 μL of pre-prepared ATP detection working solution were added to each well of a 96-well plate. The plate was gently shaken using the microplate reader’s oscillation function for 2 min to ensure thorough mixing. Subsequently, the plate was incubated at room temperature for 10 min. The relative luminescence units (RLU) were then measured to assess ATP levels.

### Mitochondrial membrane potential detection

Mitochondrial membrane potential (ΔΨm) was assessed using JC-1 and JC-10 dyes. For JC-1 staining, cells were incubated with a pre-prepared JC-1 working solution in a constant-temperature incubator for 40 min. After incubation, cells were washed twice with HBSS and observed under a fluorescence microscope using an Imaging Buffer Solution. For JC-10 detection, cells were washed with PBS and incubated in a 96-well plate with a mixture of cell culture medium and JC-10 working solution (1:1) for 20 min. After incubation, the JC-10 dye buffer was diluted and kept in an ice bath. Cells were then washed twice with JC-10 staining buffer, and 100 μL of cell culture medium was added to the reading plate. The mitochondrial membrane potential was quantified by calculating the red-to-green fluorescence ratio based on the readings.

### Western blot

HepG2 and Huh7 cells were seeded in 6-well plates (1.2 × 10^4^ cells per well) and treated with EAO (0, 50, or 100 μg/mL) for 48 h. Total protein was extracted from the cells using RIPA buffer containing protease inhibitors. Protein concentration was measured using the Pierce BCA method. Protein samples were separated by 8%–12% SDS-PAGE, and the proteins were transferred onto nitrocellulose membranes. After blocking with 5% skim milk, the membranes were incubated with primary antibodies overnight at 4 °C. The membranes were then incubated with a horseradish peroxidase-conjugated secondary antibody for 1 h at room temperature. Protein bands were visualized using the Odyssey Software Version 5.2 (LI-COR Biosciences).

### Statistical analysis

Data analysis was performed using GraphPad Prism 8.4.3. A t-test was used to analyze differences between two groups, while one-way ANOVA was used to compare multiple groups. Data is expressed as mean ± SD, with all experiments repeated more than three times. A p-value <0.05 was considered statistically significant, while no significant difference was noted when p ≥ 0.05.

## Results

### EAO Treatment Inhibits HepG2 and Huh7 cells proliferation

**FIGURE 1:**
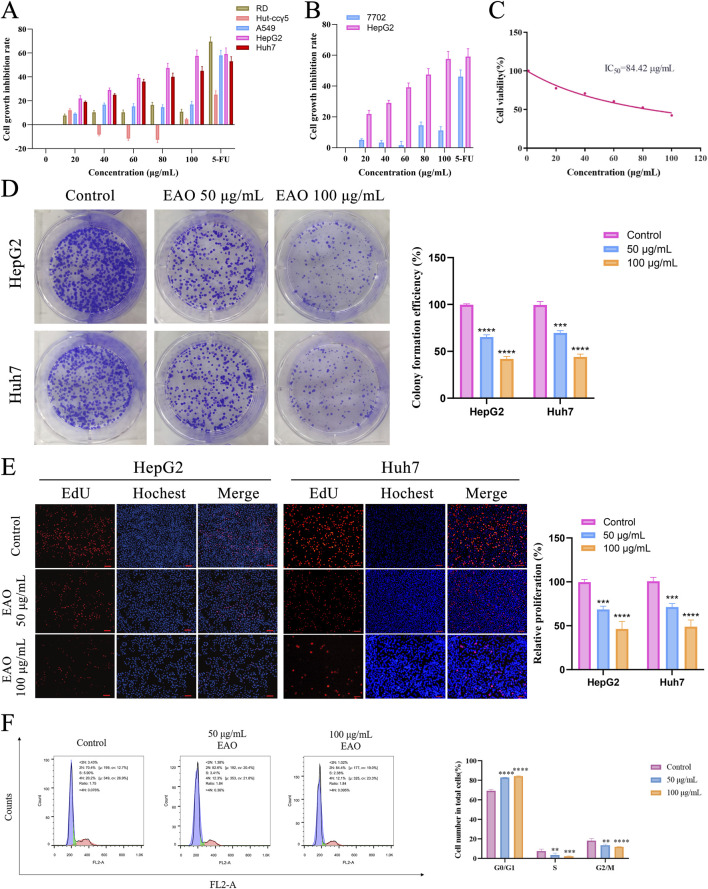
EAO Treatment Inhibits HepG2 and Huh7 Cell Proliferation **(A)** Dose-dependent inhibition of cell viability by various concentrations of EAO (0, 20, 40, 60, 80, and 100 μg/mL) compared to 5-FU (200 μM) across different cancer cell lines **(B)** Comparative analysis of the inhibitory effects of increasing EAO concentrations on healthy hepatocytes (7702) versus hepatoma cells (HepG2) **(C)** Determination of the IC50 value for EAO in HepG2 cells **(D)** Results from colony formation assays demonstrate the reduction in the number of colonies formed following EAO treatment, along with quantification of monoclonal colony counts **(E)** Representative EdU staining images of HepG2 and Huh7 cells (scale bar = 50 μm), with relative proliferation expressed as a percentage **(F)** Flow cytometric analysis of the cell cycle distribution in HepG2 cells treated with EAO. Data represent n ≥ 3 per group. Statistical significance: **p < 0.01; ***p < 0.001; ****p < 0.0001.

To assess the anti-proliferation potential of EAO, we conducted dose-response studies on multiple cancer cell lines (RD, Hut-ccγ5, A549, HepG2, and Huh7) using CCK-8 assays. Notably, HepG2 exhibited marked sensitivity to EAO treatment, showing a concentration-dependent inhibition of cell proliferation (Figure 1A). Importantly, parallel treatments of normal human hepatocytes (7702 cell line) with identical EAO concentrations (0–100 μg/mL) demonstrated no significant cytotoxicity, confirming the selective anti-cancer activity of EAO (Figure 1B). Quantitative analysis revealed an IC50 value of 84.42 μg/mL for EAO in HepG2 cells (Figure 1C). We selected 0, 50, and 100 μg/mL EAO for subsequent experiments. Colony formation assays showed that both 50 and 100 μg/mL EAO treatments significantly reduced both the number and size of HepG2 and Huh7 colonies compared to untreated controls (Figure 1D). This anti-proliferative effect was further validated by EdU incorporation assays, which demonstrated decreased DNA synthesis in EAO-treated cells (Figure 1E). Mechanistic investigations through flow cytometry revealed that EAO treatment induced cell cycle arrest at the G0/G1 phase (Figure 1F). These results collectively indicate that EAO effectively inhibits HepG2 cell proliferation through cell cycle disruption, while sparing normal hepatocytes.

### EAO induces apoptosis and mitochondrial dysfunction in HepG2 cells

**FIGURE 2:**
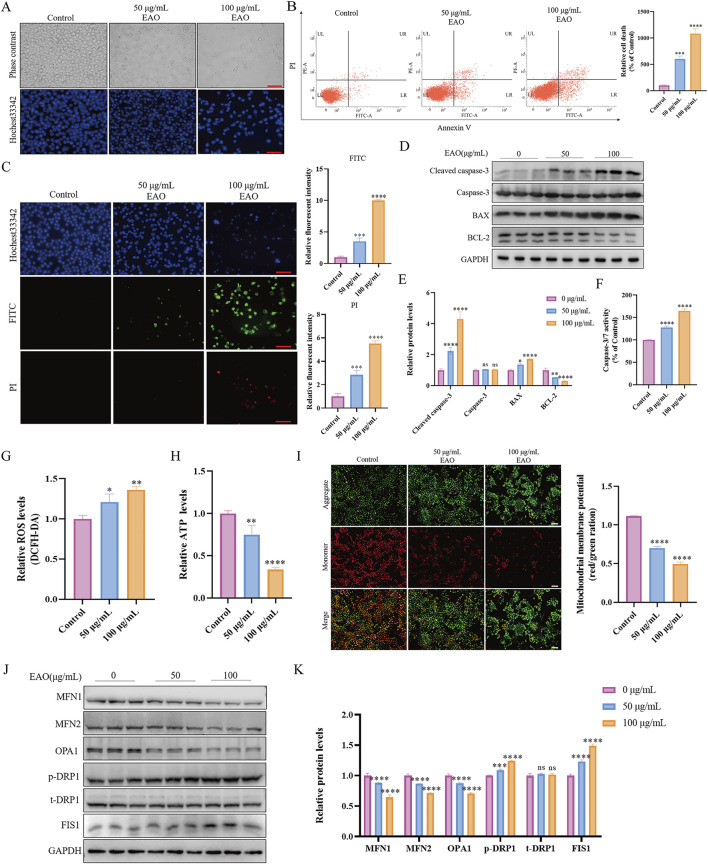
EAO Induces Apoptosis and Mitochondrial Dysfunction in HepG2 Cells **(A)** Morphological changes in HepG2 cells following EAO treatment, including nuclear condensation and membrane shrinkage (scale bar, 50 μm) **(B)** Quantification of apoptotic cells by flow cytometry using Annexin V/FITC-PI double staining **(C)** Representative fluorescence microscopy images of Annexin V/FITC-PI–stained cells (scale bar, 50 μm) **(D)** Western blot analysis of apoptosis-related proteins (e.g., cleaved caspase-3, BAX, BCL-2) **(E)** Quantification of protein expression normalized to GAPDH **(F)** Caspase-3/7 activity assay showing enhanced caspase activation after EAO treatment **(G)** Intracellular reactive oxygen species (ROS) levels following EAO exposure, indicating oxidative stress **(H)** Measurement of ATP levels reveals a concentration-dependent decline upon EAO treatment **(I)** JC-1 staining images demonstrating decreased mitochondrial membrane potential (Δψm) in response to EAO (scale bar, 200 μm) **(J)** Western blot analysis and quantification of mitochondrial dynamics–related proteins, including MFN1, MFN2, OPA1, p-DRP1, and FIS1 **(K)** Quantification of protein expression and normalized to GAPDH. Data represent mean ± SD from n ≥ 3 independent experiments. Ns: not significant; *p < 0.05; **p < 0.01; ***p < 0.001; **p < 0.0001.

Apoptotic cell death is characterized by distinct morphological and biochemical alterations. Upon EAO treatment, HepG2 cells exhibited classical apoptotic features, including nuclear condensation (pyknosis), chromatin fragmentation, and cell shrinkage (Figure 2A). Flow cytometric analysis using Annexin V-FITC/PI staining demonstrated a dose-dependent increase in apoptotic cell populations (Figure 2B). Notably, EAO at 100 μg/mL not only significantly induced apoptosis but also triggered secondary necrosis, as evidenced by double-positive Annexin V and PI staining (Figure 2C). To elucidate the underlying molecular mechanisms, we assessed the expression of key apoptotic regulators by Western blot. EAO treatment markedly increased the levels of cleaved caspase-3 and pro-apoptotic BAX, while reducing the expression of anti-apoptotic BCL-2 (Figures 2D,E). Caspase-3/7 enzymatic activity was also significantly elevated in a concentration-dependent manner, indicating activation of the intrinsic apoptotic pathway (Figure 2F).

Given the critical role of mitochondria in regulating intrinsic apoptosis, we further investigated EAO’s effects on mitochondrial homeostasis. DCFH-DA staining revealed a 2.8-fold increase in intracellular ROS levels after 24 h of EAO exposure (Figure 2G), indicating oxidative stress. This was accompanied by a substantial reduction in ATP levels (32% and 67% decrease at 50 and 100 μg/mL, respectively; Figure 2H), suggesting impaired mitochondrial bioenergetics. JC-1 staining demonstrated significant mitochondrial membrane potential (ΔΨm) collapse, with a 4.1-fold increase in the monomer/aggregate fluorescence ratio at 100 μg/mL EAO (Figure 2I). Moreover, Western blot analysis showed that EAO suppressed mitochondrial fusion proteins MFN1, MFN2, and OPA1, while enhancing the fission markers p-DRP1 and FIS1 (Figures 2J,K).

Collectively, these results indicate that EAO induces intrinsic apoptosis in HepG2 cells by promoting mitochondrial dysfunction, characterized by ROS overproduction, ATP depletion, loss of ΔΨm, and imbalanced mitochondrial dynamics. These mitochondrial alterations likely serve as upstream signals contributing to the activation of caspase-dependent cell death.

### EAO suppresses autophagic flux in HepG2 cells through LC3 processing inhibition

**FIGURE 3:**
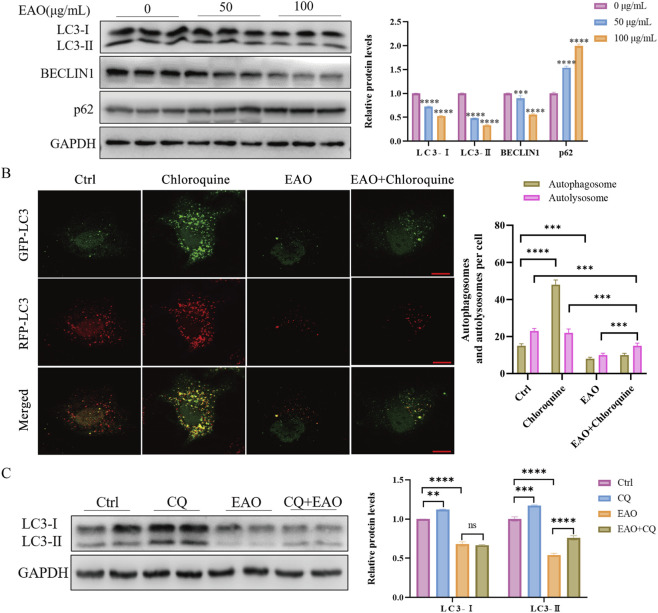
EAO Inhibits Autophagy in HepG2 Cells **(A)** Western blot analysis showing the expression of autophagy-related proteins following EAO treatment **(B)** Immunofluorescence images of LC3 expression after chloroquine treatment (EAO at IC50: 84.42 μg/mL; scale, 200 μm) **(C)** Quantitative analysis of LC3 protein levels in cells treated with EAO, with or without chloroquine. Data represent n ≥ 3 per group; ns: not significant; **p < 0.01; ***p < 0.001; ****p < 0.0001.

To further elucidate the impact of EAO on HepG2 cells’ survival mechanisms, we systematically investigated its effects on autophagy regulation in HepG2 cells. Western blot analysis of critical autophagy markers demonstrated that EAO treatment significantly downregulated LC3-II conversion while increasing p62 accumulation and decreasing BECLIN1 expression (Figure 3A), indicative of impaired autophagic activity. To distinguish between autophagy inhibition and flux blockade, we employed chloroquine (CQ), a lysosomal inhibitor that prevents autophagosome degradation. CQ treatment alone induced characteristic yellow puncta accumulation (merged red/green signals) due to impaired autophagosome-lysosome fusion. Strikingly, EAO treatment substantially diminished both red fluorescence intensity and total autophagic puncta. While CQ co-treatment partially restored red fluorescence signals (Figure 3B), this rescue effect remained limited, suggesting fundamental disruption of autophagosome formation rather than mere flux inhibition. Molecular characterization revealed that while CQ treatment (as expected) caused LC3-II accumulation through lysosomal inhibition, EAO treatment consistently reduced LC3-II levels regardless of CQ co-treatment (Figure 3C). These findings collectively demonstrate that EAO suppresses intracellular autophagy by specifically impairing the LC3-I to LC3-II conversion process, a critical step in autophagosome membrane formation and elongation.

### Prediction of targets and signaling pathways of EAO

**FIGURE 4:**
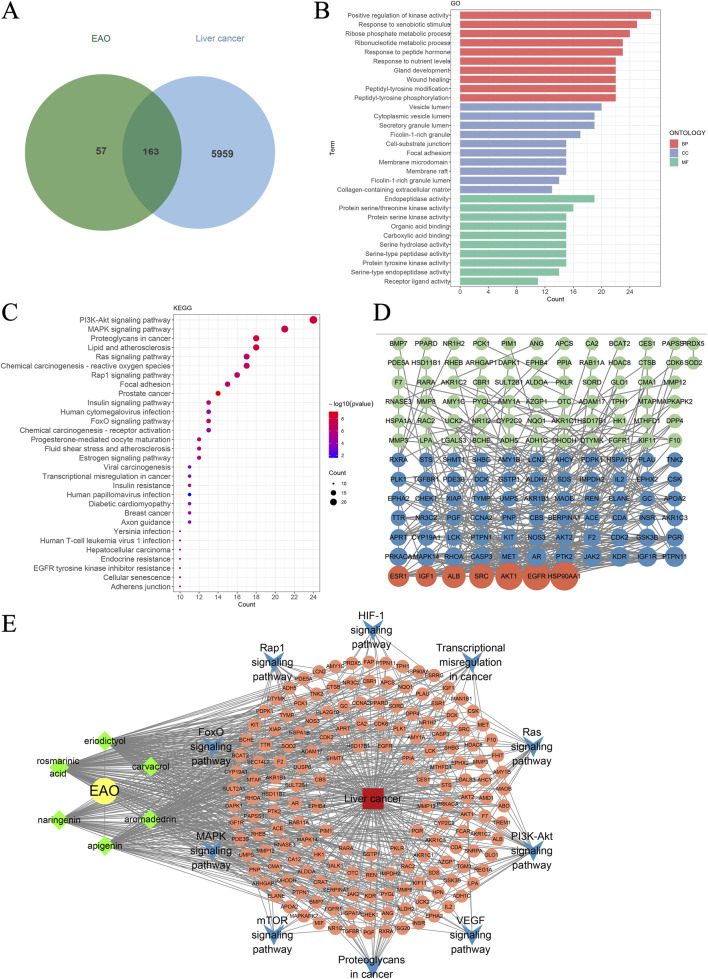
Integrated Network Pharmacology and RNA-Seq Analysis **(A)** Venn diagram illustrating that, among 220 potential EAO targets and 6,122 liver cancer-related genes, 163 key intersecting targets were identified (p < 0.05) **(B)** Top 10 Gene Ontology (GO) terms from enrichment analysis of EAO targets for liver cancer treatment **(C)** Top 30 KEGG pathways associated with EAO therapeutic targets in liver cancer **(D)** Protein-protein interaction (PPI) network constructed for the identified targets **(E)** Comprehensive “Component-Target-Pathway” network diagram.

A total of 6,122 genes related to liver cancer were identified through a keyword search in GeneCards, UALCAN, and DisGeNET databases. Intersection analysis between these genes and the predicted targets of EAO’s active components identified 163 common genes (Figure 4A). To explore the potential mechanisms of EAO in liver cancer, we performed Gene Ontology (GO) and KEGG pathway enrichment analyses. GO enrichment analysis revealed that the targeted genes were primarily involved in kinase activity and responses to external stimuli (Figure 4B). KEGG analysis showed that EAO may exert its effects on liver cancer through key pathways such as the PI3K-AKT and MAPK signaling pathways (Figure 4C). Next, a protein–protein interaction (PPI) network was constructed in Cytoscape based on STRING data for these 163 targets, revealing several hub proteins with high connectivity that may serve as critical mediators of EAO’s activity (Figure 4D). Finally, integration of active component–target relationships with enriched pathways yielded a comprehensive “Component-Target-Pathway” network. This network highlights which EAO constituents are linked to specific targets and downstream signaling cascades, providing a visual framework for EAO’s multi-targeted mechanism in liver cancer (Figure 4E). These findings suggest that EAO might modulate tumor growth and survival through these pathways.

### RNA-seq analysis of HepG2 cells treated with EAO

**FIGURE 5:**
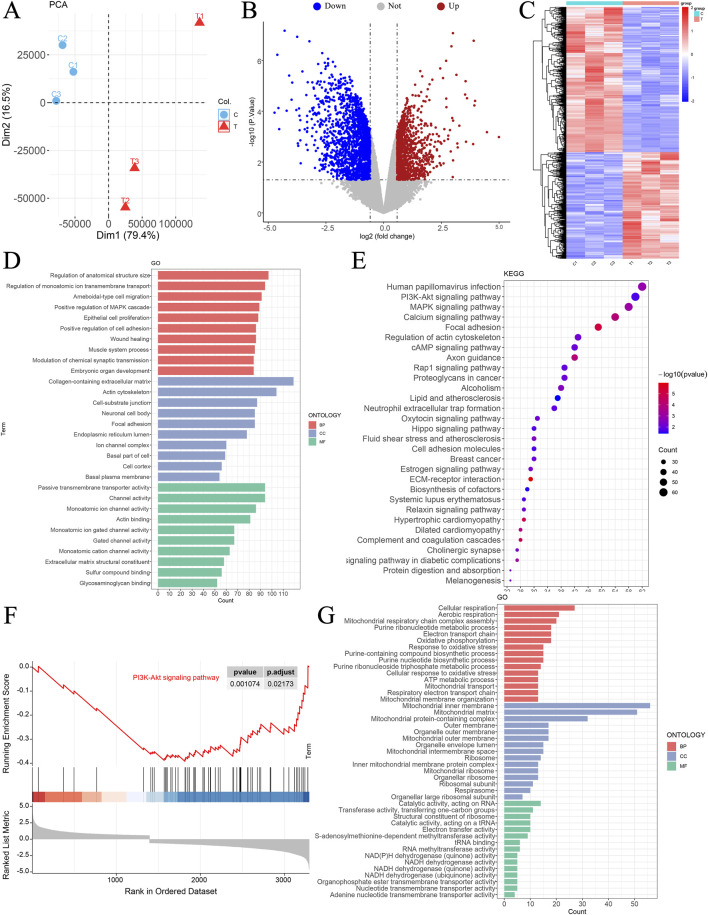
RNA-Seq Analysis of HepG2 Cells Treated with EAO **(A)** Principal Component Analysis (PCA) plot showing distinct clustering between the control and EAO-treated groups **(B)** Volcano plot indicating the distribution of differentially expressed genes between control and treatment groups **(C)** Heatmap displaying gene clustering based on differential expressions **(D)** Top 10 enriched GO terms between the control and treatment groups **(E)** Top 30 enriched KEGG pathways identified in control vs. treatment groups **(F)** Gene Set Enrichment Analysis (GSEA) illustrating alterations in the PI3K/AKT signaling pathway **(G)** Top 15 GO terms for mitochondria-related genes altered by EAO treatment (*p* < 0.05).

To further explore the molecular mechanisms underlying EAO’s effects on HepG2 cells, RNA sequencing analysis of EAO-treated HepG2 cells was performed. Principal component analysis demonstrated clear segregation between control and EAO-treated groups (PC1 = 68.3%, PC2 = 19.1%), confirming robust treatment-induced transcriptional changes (Figure 5A). Comparative analysis identified 1,248 significantly dysregulated genes (|log2FC| > 1, FDR <0.05), 682 upregulated and 566 downregulated genes. Volcano plot visualization highlighted key modulated genes (Figure 5B), while hierarchical clustering of the top 50 DEGs showed distinct expression profiles between groups (Figure 5C). Integrative pathway analysis revealed that the most significantly affected pathways were the PI3K/AKT and MAPK signaling pathways (*p* < 0.05) (Figures 5D,E). Notably, Gene Set Enrichment Analysis (GSEA) confirmed significant downregulation of PI3K/AKT signaling components (NES = −2.31, FDR q < 0.001), with suppression of AKT1/2/3 isoforms, OXPHOS gene and mitochondrial function impairment related effector proteins (Figure 5F). Since mitochondria play a central role in maintaining cellular homeostasis and are involved in apoptosis and autophagy, we conducted GO enrichment analysis of mitochondria-related genes affected by EAO treatment. The results showed that mitochondrial changes were primarily related to cell respiration, electron transport chain activity, and oxidative phosphorylation. Cellular component analysis revealed that the most significant alterations occurred in the mitochondrial inner membrane and mitochondrial matrix (Figure 5G). These findings suggest that EAO treatment may disrupt mitochondrial function, contributing to its antiproliferative effects on HepG2 cells.

### EAO inhibits the PI3K/AKT signaling pathway in HepG2 and Huh7 cells

**FIGURE 6:**
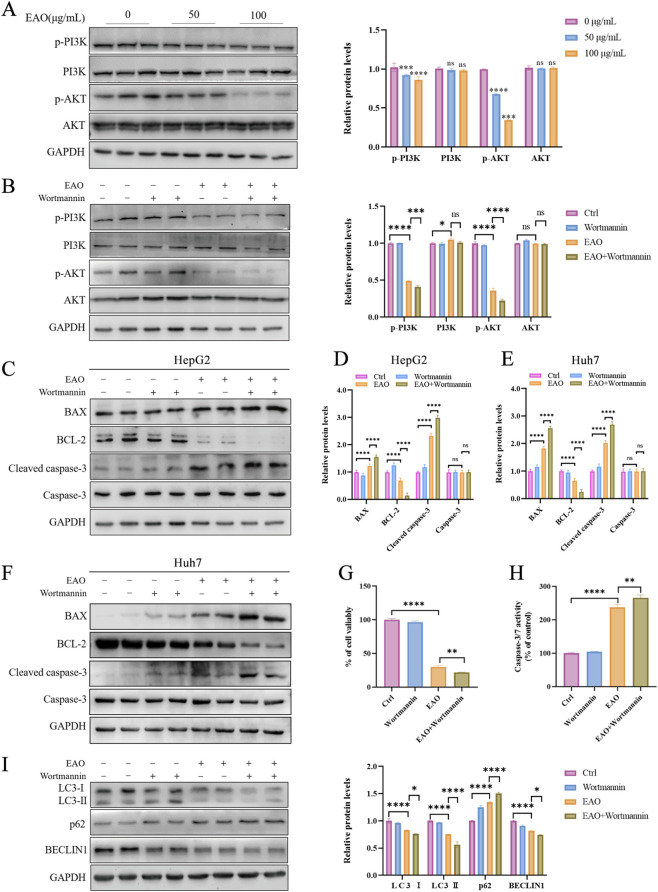
EAO Suppresses PI3K/AKT Signaling and Synergizes with Wortmannin to Promote Apoptosis and Autophagy Blockade in HepG2 and Huh7 Cells **(A)** Western blot analysis showing the suppression of phosphorylated PI3K and AKT proteins in HepG2 cells following EAO treatment. Quantification of PI3K/AKT signaling components revealing a significant reduction in phosphorylation levels **(B)** Pre-treatment with the PI3K inhibitor Wortmannin (100 nM) further decreased phosphorylated PI3K and AKT expression levels, as shown by Western blot **(C–F)** Expression of apoptosis-related proteins was further enhanced in the combination treatment group (EAO + Wortmannin) **(G)** Cell viability assay demonstrates a further reduction in survival upon combined treatment **(H)** Caspase-3/7 activity assay indicating greater enzymatic activation following co-treatment **(I)** Western blot analysis of autophagy markers showing increased p62 accumulation and decreased BECLIN1 and LC3-II/I levels in the combined treatment group (EAO at IC50: 84.42 μg/mL). Data represent n ≥ 3 per group; ns: not significant; *p < 0.05; ***p < 0.001; ****p < 0.0001. NAC reversed EAO-induced apoptosis and autophagy inhibition.

Integrated network pharmacological and transcriptomic analyses identified the PI3K/AKT signaling pathway as a critical mediator of EAO’s biological effects. Western blot analysis demonstrated that 48-h EAO treatment did not alter total PI3K and AKT protein levels but significantly reduced their phosphorylated forms (p-PI3K and p-AKT), indicating selective pathway inhibition (Figure 6A). To further validate the functional role of PI3K/AKT suppression in EAO’s mechanism, we pre-treated HepG2 cells with Wortmannin, a potent PI3K inhibitor. As expected, Wortmannin synergized with EAO, further diminishing p-PI3K and p-AKT levels (Figure 6B), confirming enhanced pathway blockade. Consistent with PI3K/AKT’s anti-apoptotic role, Wortmannin pre-treatment augmented EAO-induced apoptosis, as evidenced by increased cleaved caspase-3 and BAX levels and decreased BCL-2 expression in HepG2 and Huh7 cells (Figures 6C–F). Correspondingly, cell viability assays and Caspase-3/7 activity measurements indicated that Wortmannin further reduced cell survival and significantly upregulated Caspase-3/7 activity (Figures 6G,H).

PI3K/AKT signaling also regulates autophagy, and its suppression by EAO was amplified by Wortmannin, the autophagy flux which including the autophagy initiation and autophagosome formation was inhibited as shown by increased p62 and decreased BECLIN1 and LC3-II/I ration (Figure 6I). These results collectively demonstrate that EAO exerts its anti-tumor effects, at least in part, through PI3K/AKT pathway suppression, leading to apoptosis induction and autophagy inhibition. Pharmacological PI3K blockade (Wortmannin) synergizes with EAO, reinforcing its therapeutic potential.

**FIGURE 7:**
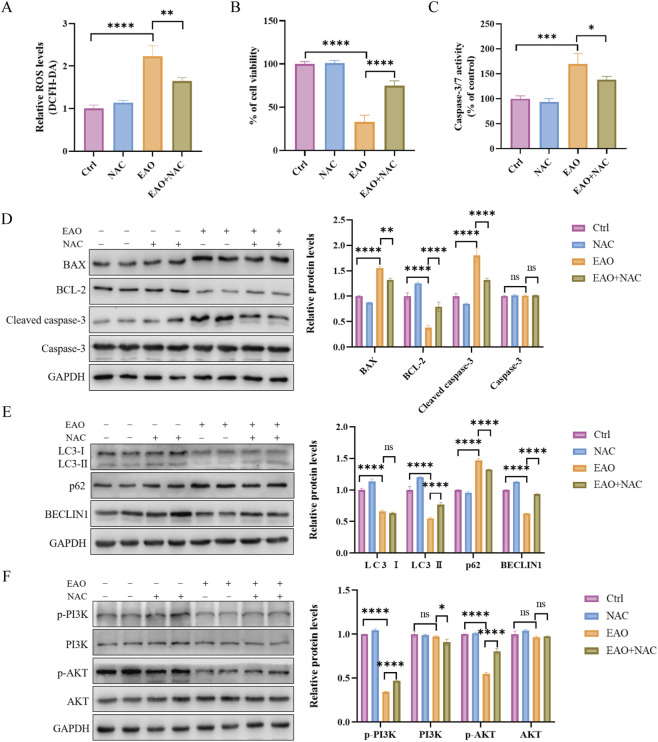
NAC Reverses EAO-Induced Apoptosis and Autophagy Inhibition **(A)** Measurement of ROS levels demonstrates that NAC treatment reduces ROS induced by EAO **(B)** Cell viability assay indicating that NAC ameliorates the decrease in viability caused by EAO **(C)** Caspase-3/7 activity analysis showing that NAC mitigates the EAO-induced increase in activity **(D)** Western blot analysis of apoptosis-related proteins illustrating that NAC decreases cleaved caspase-3 and BAX levels while increasing BCL-2 expression **(E)** Analysis of autophagy markers revealing that NAC reverses EAO-induced changes in p62, BECLIN1, and LC3-II/I expression **(F)** Western blot analysis showing that NAC treatment partially restores phosphorylated PI3K and AKT levels. EAO was used at its IC50 concentration of 84.42 μg/mL. Data represent n ≥ 3 per group; ns: not significant; *p < 0.05; ***p < 0.001; ****p < 0.0001.

To determine whether ROS mediates EAO-induced apoptosis and autophagy inhibition, we treated EAO-exposed HepG2 cells with the ROS scavenger N-acetylcysteine (NAC). EAO significantly elevated intracellular ROS levels, which were effectively suppressed by NAC (Figure 7A). Subsequent experiments demonstrated that NAC treatment markedly reversed EAO-induced cytotoxicity (Figure 7B) and Caspase-3/7 activation (Figure 7C). Western blot analysis further revealed that NAC downregulated pro-apoptotic markers (cleaved caspase-3 and BAX) while restoring anti-apoptotic BCL-2 expression. Additionally, NAC counteracted EAO’s effects on autophagy, reducing p62 accumulation and restoring BECLIN1 and LC3-II/I levels (Figures 7D,E). Notably, NAC partially rescued PI3K and AKT phosphorylation (Figure 7F), indicating that ROS plays a pivotal role in EAO-induced suppression of the PI3K/AKT pathway, ultimately leading to apoptosis induction and autophagy inhibition.

## Discussion

**FIGURE 8:**
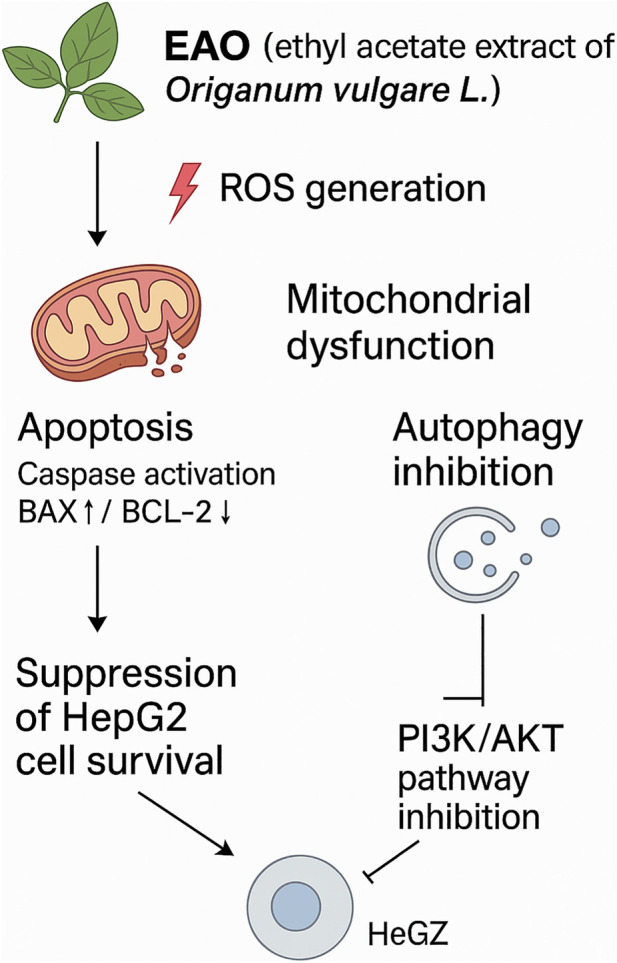
Graphical abstract of oregano extract induces apoptosis and inhibits autophagy in HepG2 cells via the PI3K/AKT Pathway. EAO combats hepatocellular carcinoma through mitochondrial dysfunction, induction of apoptosis, inhibition of autophagy, and ROS-dependent suppression of the PI3K/AKT pathway.

This study demonstrates that the EAO exerts anti-hepatocellular carcinoma effects through the induction of mitochondrial dysfunction, apoptosis, and inhibition of autophagy. Mechanistically, EAO suppresses the PI3K/AKT pathway in a ROS-dependent manner (Figure 8). These findings highlight EAO as a promising low-toxicity candidate for the treatment of liver cancer. (Collier et al., 2023; Rossmann et al., 2021). A key finding is that EAO elevates intracellular ROS, which disrupts mitochondrial integrity by damaging critical proteins and lipids, leading to reduced ATP production and loss of mitochondrial membrane potential (ΔΨm) (Shao et al., 2021; Su et al., 2023). This dysfunction was further evidenced by an imbalance in mitochondrial dynamics, characterized by downregulation of fusion proteins (MFN1, MFN2, OPA1) and upregulation of fission mediators (p-DRP1, FIS1) (Wang et al., 2023). Such dysregulation destabilizes mitochondrial homeostasis, priming cells for apoptosis, and suggesting that EAO targets mitochondrial integrity as a key strategy to trigger programmed cell death.

Beyond mitochondrial disruption, EAO inhibits the PI3K/AKT pathway, a central regulator of cell survival and autophagy (Liu et al., 2023; Gupta et al., 2021; Yu et al., 2022; Martini et al., 2014; Biswas et al., 2024; Khan et al., 2018; Fitzwalter et al., 2018). The marked reduction in p-PI3K and p-AKT levels indicates pathway suppression, aligning with the observed pro-apoptotic and anti-autophagic effects (Hama et al., 2023; Turdo et al., 2021; Yuan et al., 2023). Network pharmacology and RNA-Seq analyses corroborated these findings, identifying PI3K/AKT signaling as a major target of EAO and revealing its impact on related gene networks in liver cancer (Shang et al., 2023; Zhu et al., 2021; Liu et al., 2024). However, the best way to measure the autophagy activity is by doing autophagic flux (Klionsky et al., 2016; Klionsky et al., 2021), we will conduct further experimental validation in subsequent studies. Moreover, the use of Wortmannin supports the involvement of PI3K/AKT signaling. However, the observed enhancement by an inhibitor does not conclusively establish that suppression of PI3K/AKT represents the primary mechanism. In our subsequent studies, we will use AKT overexpression experiments, constitutively active AKT rescue experiments, or other PI3K/AKT activators to determine whether pathway restoration can alleviate EAO-induced apoptosis, thereby further elucidating the mechanisms underlying EAO.

These results are consistent with prior reports on carvacrol and flavonoids, which exhibit anticancer activity via ROS generation and PI3K/AKT modulation (Sharifi-Rad et al., 2018). Notably, EAO demonstrated selective cytotoxicity against HepG2 cells, suggesting a favorable therapeutic window.

The findings hold significant clinical relevance, particularly for advanced liver cancer, where current therapies face challenges such as drug resistance, recurrence, and poor efficacy (Huang et al., 2022). EAO’s dual ability to induce apoptosis and block autophagy in HepG2 cells, coupled with its low toxicity profile, positions it might as a promising multi-targeted therapeutic candidate. However, limitations must be acknowledged. *In vitro* models may not fully recapitulate tumor complexity. EAO’s multi-component nature could lead to variability in composition and bioactivity. Future studies should focus on validating these findings *in vivo* models and exploring optimal dosing strategies. Moreover, investigating the synergistic potential of EAO in combination with existing chemotherapeutics or targeted therapies could further enhance its clinical applicability.

## Conclusion

In summary, EAO suppresses HepG2 proliferation by inducing mitochondrial dysfunction, activating apoptosis, and inhibiting autophagy via ROS-dependent PI3K/AKT pathway suppression. The integration of network pharmacology and transcriptomics provided robust mechanistic insights, advancing our understanding of EAO’s anticancer properties. These findings underscore the potential of natural-product-derived therapies for liver cancer and warrant further investigation into EAO’s clinical applicability.

## Data Availability

The original contributions presented in the study are included in the article/supplementary material, further inquiries can be directed to the corresponding author/s. All raw sequencing reads, alignment files and processed expression matrices have been deposited in the Genome Sequence Archive (GSA) of the China National Center for Bioinformation (CNCB) under the accession PRJCA067345.
